# Modelling auditory attention

**DOI:** 10.1098/rstb.2016.0101

**Published:** 2017-02-19

**Authors:** Emine Merve Kaya, Mounya Elhilali

**Affiliations:** Laboratory for Computational Audio Perception, Department of Electrical and Computer Engineering, The Johns Hopkins University, 3400 N Charles Street, Barton Hall, Baltimore, MD 21218, USA

**Keywords:** computational model, auditory attention, auditory scene, bottom-up, top-down, salience

## Abstract

Sounds in everyday life seldom appear in isolation. Both humans and machines are constantly flooded with a cacophony of sounds that need to be sorted through and scoured for relevant information—a phenomenon referred to as the ‘cocktail party problem’. A key component in parsing acoustic scenes is the role of attention, which mediates perception and behaviour by focusing both sensory and cognitive resources on pertinent information in the stimulus space. The current article provides a review of modelling studies of auditory attention. The review highlights how the term attention refers to a multitude of behavioural and cognitive processes that can shape sensory processing. Attention can be modulated by ‘bottom-up’ sensory-driven factors, as well as ‘top-down’ task-specific goals, expectations and learned schemas. Essentially, it acts as a selection process or processes that focus both sensory and cognitive resources on the most relevant events in the soundscape; with relevance being dictated by the stimulus itself (e.g. a loud explosion) or by a task at hand (e.g. listen to announcements in a busy airport). Recent computational models of auditory attention provide key insights into its role in facilitating perception in cluttered auditory scenes.

This article is part of the themed issue ‘Auditory and visual scene analysis’.

## 1. Introduction

While at a cocktail party, we often find ourselves flooded by a cacophony of sounds that impinge on our ears from a multitude of sources. The challenge of directing our attention despite numerous prominent distractors, referred to as the ‘cocktail party problem’ [1,2], engages intricate neural networks and cognitive processes that enable the brain to parse information in the environment [3]. These processes allow us to navigate our surroundings, focus on conversations of interest, enjoy the background music and be alert to any salient sound events such as someone calling our name or the ringtone of our phone. Throughout this scene analysis process, attention plays a crucial role in mediating both perception and behaviour by focusing both sensory and cognitive resources on pertinent information in the stimulus space [4]. This article provides a review of modelling studies of auditory attention and their impact on studies of attention in audition.

Attention is not a single, unidirectional process [5,6]. It can be modulated by ‘bottom-up’ stimulus-driven factors, as well as ‘top-down’ task-specific goals, expectations and learned schemas. Ultimately, attention is a form of information bottleneck that samples the massive sensory input constantly impinging on our ears and directs sensory and cognitive resources to the most relevant events in the soundscape [7]. Owing to the complexity of auditory scenes, the relevance of a sound event can be dictated by the scene itself (e.g. a conspicuous sound event such as a gunshot that would attract attention) or by a task at hand (e.g. to follow a conversation with a friend amidst competing sound sources).

While attention has started to garner increasing interest from the auditory research community [8–10], there is not much tradition of developing computational models of attention in the context of sound systems. Such models would need to account for the auditory system's ability to adapt to the demands of an ever-changing acoustic environment and task goals. Recent physiological findings have been amending our views of processing in the auditory system, replacing the conventional view of ‘static’ processing in sensory cortex with a more ‘active’ and malleable mapping that rapidly adapts to the tasks at hand, sound context and listening conditions [11]. Numerous studies have revealed that our auditory experiences can have significant local effects by transforming receptive field properties of individual neurons, and profound global effects by reshaping cortical circuits [12,13]. These effects extend beyond early sensory areas and indicate attentional modulation throughout the auditory cortex, shedding light on the distributed nature of processing in auditory pathways in the context of cocktail party settings [14].

Although research on the neural underpinnings of these networks is thriving, our understanding of the exact role of adaptive stimulus- or task-directed processing remains in its infancy. The field is particularly challenged by the lack of theories that integrate our knowledge of cortical circuitry in the auditory pathway with adaptive and cognitive processes that shape behaviour and perception of complex acoustic scenes. By contrast, active and adaptive processing has more commonly been explored in models of the visual system. These implementations typically model predictive coding in the visual thalamus (LGN), contextual modulation in primary visual cortex (V1), attentional modulation in higher cortical areas (V2 and V4, area MT) and decision-making in parietal and frontal cortex [15,16]. That being said, recent theoretical studies are providing insight into common processing traits of active attention across modalities [17].

A number of perspectives have emerged about conceptual frameworks for understanding the role of attention in auditory perception. Much of this work closely parallels theories from vision in which attention is viewed as a multifaceted phenomenon that encompasses mechanisms of selection, integration and sampling [18]. In one view, attention can be considered as a filtering or a selection mechanism. This interpretation ties in directly with findings of receptive field properties in sensory cortex, whereby neurons can be viewed as filters whose properties are modulated by task-directed attention and whose activity can be adapted depending on sensory contexts [11,19]. At a larger scale, this view extends to object-based or semantic selection processes whereby attention to a specific target or class of sounds (e.g. speech, music) would engage specific neural circuits [14,20]. This view parallels selection theories in vision, which present frameworks for funnelling only relevant information to the processing pipeline, either at an early or later stage, acting as an informational bottleneck that mitigates the limited computational resources of the sensory system [5]. An alternative view of attention frames it as an integration mechanism, whereby attentional feedback acts as a prior to bias processing of certain stimuli of interest. Many theories of sound perception in complex settings favour this view, under which attention operates as a ‘glue’ that binds together elements belonging to the same event. This interaction between object formation and selective attention is instrumental in guiding the organization of the foreground and background, and the interaction between the perceptual representations of sound targets and interferers [21,22].

**Figure 1.**
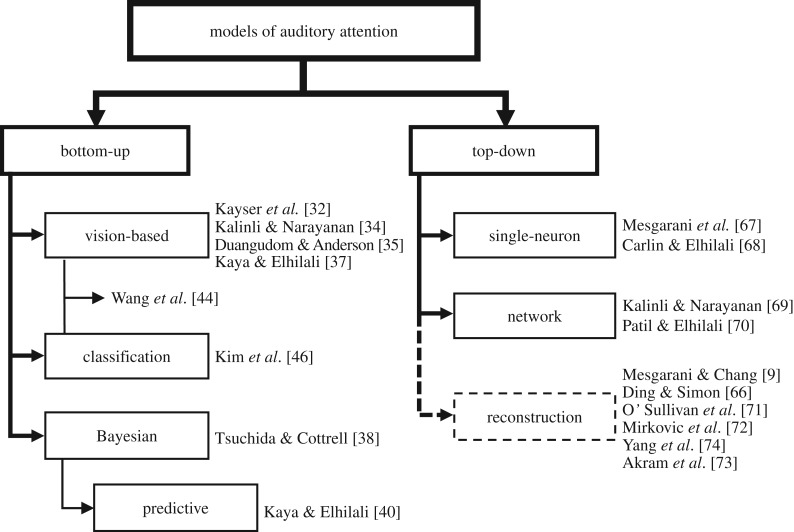
A broad classification of models described in this review. Reconstruction techniques are not computational models in the traditional forward architecture of sound to ‘perception’; however, this methodology provides valuable insight in understanding task-directed attention.

The present review aims to provide a synopsis of current computational efforts in modelling attention in the context of auditory scene analysis. Figure 1 provides a general overview of models included in this review. These models often cluster around accounts of bottom-up or top-down processing, though they remain confined by hand-picked experimental observations. The article reviews the relevant perspectives for both sensory- and task-driven attentional models and discusses some efforts to validate such models. The review also touches on relevant applications of such models in audio systems and hearing technologies.

## 2. Models of auditory attention

### (a) Bottom-up attention

Models of bottom-up attention remain very scarce in the auditory literature. The limited efforts in this direction have greatly benefited from the very prolific research on bottom-up attention (or salience) in vision. Indeed, visual salience is a thriving research field that has resulted in a rich body of work examining the perceptual attributes underlying visual salience [23], as well as its behavioural correlates [24] and underlying neuroanatomy [25–27]. In parallel, computational models of visual salience have built on this knowledge and made use of the availability standardized eye-tracking datasets to develop detailed Bayesian and hierarchical accounts of visual perception [28]. These models can not only account for human behaviour in natural scenes, but are able to expand the possibilities of computer vision applications to tackle challenging visual scenes in fields such as robotics, medical imaging and surveillance systems [29–31].

Building on this tradition in the visual modality, early models of auditory bottom-up attention adapted popular visual salience models to the domain of sound. Kayser *et al*. presented one of the early efforts in this direction [32]. This work treated the time–frequency representation of sound as an ‘auditory image’ from which spectro-temporal features such as intensity and spectro-temporal contrast can be extracted to parallel the feature analysis process in vision models [33]. The back end of the model was essentially a visual salience model, in which all features were scaled to generate multi-scale maps which were then normalized to highlight conspicuous peaks and integrated to provide an auditory salience map. Though operating on relatively simple features and adopting a vision-based integration architecture, this model was able to reliably match both human and monkey behavioural responses in tasks involving detection of salient sounds embedded in different backgrounds. This work not only demonstrated that salience processing in the brain may share commonalities across sensory modalities, but it also provided a guide to designing psychoacoustical experiments to probe auditory bottom-up attention in humans.

This initial effort was later expanded to incorporate more intricate analyses of auditory features. Work by Kalinli & Narayanan [34] operated on the same auditory image and salience extraction architecture but extended the feature set to include pitch and orientation along both time and frequency, hence incorporating more relevant auditory cues. It also provided an improved contrast computation scheme to derive feature maps, making them more robust to noise and multiple salient locations. Duangudom & Anderson [35] extended the feature analysis to incorporate more biologically plausible mechanisms that mimic processing in the peripheral and central auditory system [36]. This analysis allowed the derivation of spectro-temporal modulation features that simulate neural responses in the mammalian auditory cortex. These neural-like processes provided a multi-scale mapping of the incoming auditory stimulus, effectively replacing the parallel feature maps favoured in earlier auditory salience models. While the salience analysis was similar in essence to that for vision-based models, this study began to steer the literature towards placing an emphasis on biological plausibility.

**Figure 2.**
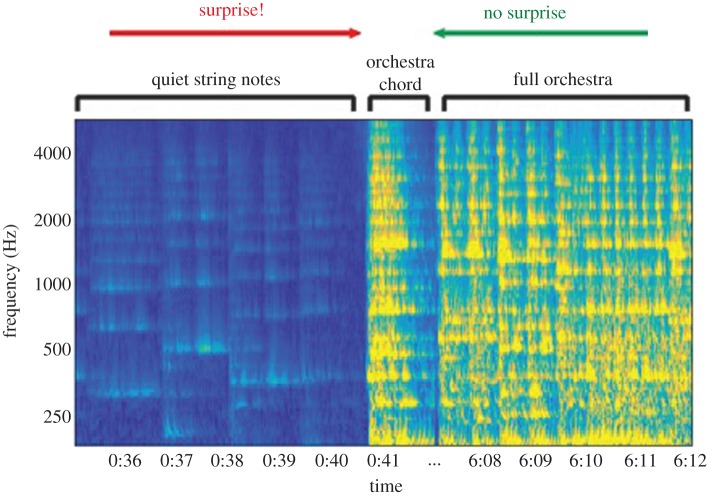
The spectrogram (time–frequency ‘image’) of an excerpt from Haydn's Surprise Symphony. Marked times correspond to the approximate location in the second movement. The surprising section is a loud chord played by the entire orchestra following a long passage of quiet string instruments. We consider the scenario of an orchestral passage immediately following the surprise chord. If the passage were reversed in time, the surprise chord would no longer be surprising, and the switch to a quiet passage is not as surprising as the switch to a sudden loud passage. This figure demonstrates the dependence of auditory salience on time and context.

Despite their relative success in extending vision-based frameworks to audition, all of the aforementioned models failed to account for an important distinction between auditory and visual processing, notably the nature of sound as a temporally evolving entity. By treating the time (*T*)–frequency (*F*) spectrogram as an auditory image, these models treated the *T*–*F* dimensions as spatial *X*–*Y* axes, failing to process the time axis as a special dimension. Effectively, the auditory image approach ignores temporal build-up and short- and long-term dependencies, and results in non-causal analyses of current events based on future information. Consider for instance a musical scene such as Haydn's Surprise Symphony (figure 2): a mellow string passage abruptly interrupted by a loud, full orchestra chord—a highly salient section. If the chord was repeated shortly after, you might be surprised again, but not as much as the first time, as you have now adjusted your expectations as to what might occur in the piece. If this chord were to start regularly repeating, it would eventually blend into the music and attract little attention. Now consider if this scene were played backwards, so the loud chord was heard repeatedly from the onset. None of the occurrences would surprise the listener—the salience has disappeared. The surprise only works when the music is considered as a temporal entity.

One of the first models to address this problem computed a temporal salience map similarly to the model of Kayser *et al*., but considered all of the features as evolving temporally, rather than as two-dimensional images [37]. The feature space was expanded to include perceptual properties of sound: loudness, pitch and timbre. All features were analysed over time to highlight their dynamic quality before normalizing and integrating across feature maps in line with vision-based models. By contrast, Tsuchida & Cottrell [38] adapted a different, statistics-based approach from the vision literature [39]. Their implementation combined long-term scene statistics computed from natural sound samples with local, rapidly changing statistics of the current incoming sound. In this framework, salience became a probabilistic account, where a sound is flagged as salient if it is determined to be unusual relative to learned statistics. This model was also the first to consider the computational efficiency of the features used, where a cochleogram was adopted instead of a spectrogram and principal component analysis was applied to reduce feature dimensionality while retaining significant variations in the features.

Even with the advances achieved by temporal salience models, basing attentional mechanisms on processes from the visual domain inherently limits the capabilities of an auditory salience model. Recognizing this, efforts in modelling auditory attention began shifting from adaptations of the visual literature to building upon inspiration from mechanisms known or hypothesized to take place in the auditory pathway. As this research area is yet in its early stages, there is an array of possible mechanisms to explore, and the following models have explored different avenues to modelling bottom-up auditory attention.

Kaya & Elhilali [40] proposed the first auditory attention model that was not based on a vision equivalent, but was rather motivated by processing known to occur in the auditory pathway. This model explored the role of predictive coding and theories of auditory deviance detection as possible underlying mechanisms determining auditory salience in the brain [41–43]. This approach puts great emphasis on the role of processing events over time and shaping neural responses of current sounds based on their preceding context. Kaya & Elhilali employed a rich feature space modelling human perception of sound [36]. This model mapped the acoustic waveform onto a high-dimensional auditory space that explicitly encoded perceptual loudness, pitch and timbre of the incoming sound, building upon evolving temporal features [37]. The attention model collected feature statistics over time and made predictions about future sensory inputs. Salient times were flagged as those for which the incoming features differed significantly from expectations. Another novel aspect of this model was the role of integration across features in guiding salience predictions. Earlier models typically employed a simple linear combination across features with a fixed weight for each feature. The Kaya & Elhilali model rejected the notion of independence across auditory features of a complex scene in guiding salience perception. Instead, the model proposed a nonlinear interaction across the feature space, implemented by asymmetrical weights between pairwise features, and guided by psychoacoustic experiments.

Two trends emerged from this work that are reflected in most current auditory attention models: building probabilistic expectations of sound to derive salience, and employing behavioural responses from perceptual experiments with human listeners to learn properties of acoustic features relevant for salience perception. The idea that salience is derived from statistics gathered over the scene was further explored in the work of Wang *et al*. [44]. This study computed Shannon entropy as a measure of the informational value of incoming sound segments, and classified them as salient or ordinary depending on whether they contained a high amount of information. This is in line with the concept that bottom-up attention alerts us to important events in a scene. Moreover, the study by Wang *et al*. offered a composite system that combined parallel paths including: (i) a temporal analysis of sound features operating on different components derived from mel-frequency cepstral coefficients [45], an alternative and very popular way to represent frequency features based on perceptual measures of pitch; (ii) a spectral mapping analysing the power spectral density of the stimulus and (iii), the image salience model based on mechanisms by Kayser *et al*. [32]. This composite system demonstrated the benefits of extending the vision-based model and provided further robustness to salience estimates especially in real noisy soundscapes.

In contrast with more theoretical approaches to auditory salience, Kim *et al*. [46] took a more data-driven approach by employing human behavioural judgements of salience to train a linear classifier that performed a simple filtering followed by feature integration based on data-driven weights. Behavioural data were gathered by subjects annotating salient locations in natural recordings of conference room meetings, and these data were used to train a model that maximized the separation between the salient and non-salient sound segments in the feature space. The results revealed that the emerging discriminant was shaped to detect temporal and frequency contrasts, and most specifically worked as an onset detector. Tordini *et al*. [47,48] approached the problem from the opposite direction: while Kim *et al*. used no prior knowledge of acoustical features to guide their feature estimation, Tordini *et al*. tested the contribution of acoustic features in defining auditory salience. Features such as temporal centroid, spectral centroid, harmonicity, effective duration and tempo were all found to correlate with salience ratings. The results also revealed interactions between some of these features in line with observations from Kaya & Elhilali [40].

It is worth highlighting that one of the challenges of studies of auditory salience is the open interpretation of what auditory salience refers to. Visual salience has historically relied on measures of eye gaze despite their shortcomings [28,49,50]. In audition, the lack of unified metrics to define salience remains a major challenge. Unmistakably salient scenarios such as a loud explosion or a male talking amongst females result in large enough loudness or pitch differences that every auditory salience model should be able to detect outlier events. However, more intricate processing is necessary for auditory events that are not as objectively salient, such as noticing a cricket among cicadas. The simple image-based features extracted in most of the aforementioned models are insufficient to capture subtle changes in temporal dynamics. Furthermore, feature interactions play an important role in determining perceived salience [40,48]—a factor unaccounted for in most models.

### (b) Top-down attention

In contrast with bottom-up attention, top-down models of auditory selective attention build on a richer body of work investigating the neural underpinnings of task-driven attention in the auditory system. It is well established that neural activity across the auditory cortex is heavily modulated by directed attention [9,13,51]. Hubel *et al*.'s early findings in the late 1950s [52] showed modulation of neural activity of single units in cat auditory cortex when animals paid attention to novel or surprising acoustic events, such as jingling of keys. They dubbed such neurons ‘attention units’ in the auditory cortex. Since then, many studies have reported similar ‘attention’ effects under controlled behavioural conditions, in different animal models and across various auditory cortex regions.

**Figure 3.**
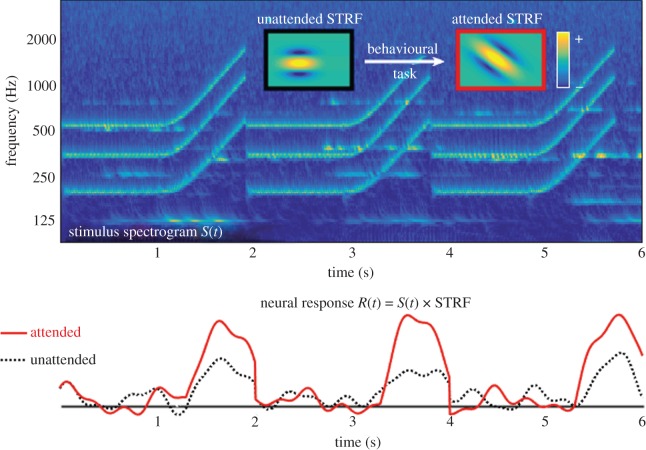
Attending to a particular sound characteristic tunes the neural spectro-temporal receptive fields (STRFs) and boosts the neural signal at times of attended event. Violin notes are overlaid with frequency modulations (FMs), illustrated with the spectrogram *S*(*t*). When instructed to attend to the FM segments, the STRF adapts to the orientation of the modulations, resulting in an enhancement in the neural response *R*(*t*).

Characterization of the tuning properties of cortical neurons using computational techniques has played a major role in investigating adaptive effects of attention on cortical activity. Specifically, spectro-temporal receptive fields (STRFs) are mathematical descriptions of the selectivity of individual neurons in response to sound events [53]. The STRF is a two-dimensional time–frequency representation of the tuning properties of cortical neurons (figure 3). From a systems theory viewpoint, each neuron can be thought of as a filter whose STRF describes the time–frequency attributes that excite the neuron [54,55]. Evidence from behaving animals revealed that as behavioural goals changed, the tuning characteristics of individual neurons as captured by their STRFs adapted rapidly [56–58]. This neural adaptation, or rapid plasticity, plays a role in enhancing neural responses to temporal and spectral modulations belonging to the target sound events, the foreground, and suppressing those that fall outside the target, the background (figure 3). Effectively, under control of attention, the neural population appears to increase the contrast between the target and background, hence facilitating focusing on sound events of interest [11]. Crucially, this process appears to be rapid, induced by attention, dependent on task and reward structure. It reflects the behavioural state of the animal [59] and spans both primary and higher auditory areas [60].

Beyond findings at the single-neuron level in animal models, various non-invasive techniques have been used to investigate the extent of attentional modulation across auditory cortex for more complex auditory scenes in human listeners. Results using functional magnetic resonance imaging and electroencephalogram (EEG) have confirmed attention-driven increase of neural activity in the auditory cortex [61,62]. Neural effects revealing distributed activity induced by spatial and non-spatial forms of auditory attention have also been observed [14,63]. Different types of attention, notably feature-based versus object-based attention, appear to induce differential activation engaging areas such as planum temporale and different regions of the superior temporal gyrus [64,65]. Tying back to results from single units in animal models, recent advances in computational methods allowed analysis of neural recordings in human listeners using magnetoencephalography (MEG) and surface electrodes that revealed greater activation to attended sounds relative to unattended sounds [9,66]. Going further, mathematical tools are now being developed to allow estimation of ensemble receptive fields from MEG and EEG recordings, laying promising groundwork to unify results across different paradigms for a complete account of selective attention processing in the brain [10].

Despite the growing body of work supporting evidence that responses across auditory cortex are modulated by attention, translating such knowledge into computational models has been slow. One avenue in modelling has been to explicitly characterize the adaptation mechanism of STRFs. Mesgarani *et al*. [67] hypothesized that the spotlight of attention works to enhance the separation between task-relevant target stimuli and the distractor background. Thus, the optimal STRF can be modelled as the filter that gives the highest discrimination between the neural responses to target and background acoustics, resulting in a deterministic linear system that can apply gain to physical features of the auditory input. In this framework, selective attention can work in a multitude of ways by enforcing different constraints based on perceptual goals, e.g. when listening for short dripping sounds to find the source of a water leak, optimization cost would be increased for slow temporal dynamics, or when attending to a male in a busy room full of children, lower pitches would be enhanced. While relatively simple, this model provides a powerful account of attentional effects at the single-neuron level. Still, it is limited in its ability to extend beyond orienting attention to physical properties of sound (e.g. attend to a class of sounds as opposed to a specific exemplar) and is invariant to task structure due to its implementation. In the last example, if the task were to ignore the male, the adaptation result would not be guaranteed to be different from that for the attend task, as the model separates two signals (male, children) without a conceptual knowledge of task demands (target/distractors).

Recognizing these limitations, Carlin & Elhilali [68] proposed a framework to account for an explicit notion of foreground and background, assigning binary labels to distinguish target sound segments from reference segments as defined by a behavioural task. The addition of task structure to the model resulted in opposite adaptation patterns when the task was switched between reward (foreground) and evasion (background), in line with observed neurophysiological responses at the level of primary auditory cortex in behaving animals [57]. The model was expanded to allow for object-based attention selection, which can ‘focus attention’ on simple abstractions based on physical properties of sound, rather than the acoustics themselves. For instance, attending to speech as a sound class (regardless of specific utterances and who the speaker is) requires ignoring details of specific acoustic instantiations and responding to abstracted representations of speech that distinguish its characteristics from those of other classes (or objects). The authors modelled such object-based selection as constraints on magnitude and phase profiles of the spectro-temporal dynamics of sound, and provided simulation results to show that modelled STRFs sharpen and orient to target modulations in line with reported physiological effects [56]. Future research is necessary to unify the feature and object-based attention models, and provide neural recording data that better account for attention to complex abstractions of sound.

Another body of work modelled selective attention in a more abstract way by incorporating the attentional gains observed in neurons across physiological experiments into computational models implementing various components of auditory scene analysis. Kalinli & Narayanan [69] extracted the ‘gist’ of an auditory scene from the biologically motivated acoustic features used in their model of salience [34], and employed a neural network to automatically learn optimal gains given specific tasks, such as scene classification. Patil & Elhilali [70] implemented the hypothesis that attention acts as a prior in a Bayesian representation of the information from the senses [7]. This model used a two-stage computational framework for recognition of acoustic scenes: a feature-extraction stage that mimicked processing in the auditory pathway from cochlea to primary auditory cortex, and an object-mapping stage that performed classification of features into scene types. Top-down attention worked at both the feature and the object level by applying gains onto the spectro-temporal filters that extract the features, and by adjusting the parameters of a scene classifier to optimize detection of the target scene.

The studies described thus far have taken a forward approach to modelling attention: given the sound input, they predict neural responses and compare the model output with brain responses. Some recent studies have taken a reverse approach to characterizing attention by reconstructing the sound input from recorded neural signals and comparing the reconstructed acoustic waveform with the input to illuminate the aspects of the soundscape that are most prominently represented in the recorded cortical area. While employing regression methods to reconstruct the sensory input from neural recordings is not new, the potential of this paradigm to study the effects of attention has only recently been used to demonstrate exciting results. Mesgarani & Chang [9] reconstructed the spectrogram of the input from intracranial recordings to show that neural representations code salient acoustic features of sound; the reconstruction correlated most strongly with spectro-temporal areas of high energy in the attended source. Further, Ding & Simon [66] reconstructed the input sound envelope from MEG recordings to show that it correlated more closely with the attended speech than the unattended speech in a scene of competing concurrent speakers. This set-up has been extended to reconstruct the attended speech from noisy single-trial EEG recordings [71,72], an especially important development for the EEG domain where noise reduction techniques coupled with averaging a high number of trials are typically necessary to estimate the neural signal. With this established framework, biologically plausible models are being designed to reconstruct the input sound from neural recordings, using dynamic state-space models [73] and deep neural networks [74], extending our understanding of attentional gain at the systems level.

## 3. Validation of auditory attention models

While eye-tracking data provides objective evaluation metrics for vision models, attention models in audition have suffered from a lack of clear salience metrics. Most attention models mentioned in this review use their own validation data and metrics, ranging in scope from single-neuron activity to human responses or carefully selected sound events or scenes with attended or salient ‘ground-truth’ determined conceptually by the experimenter. Unfortunately, there is so far little consensus on the best way to probe effects of attention on auditory perception, whether it is task-directed or purely based on salience.

With the first auditory salience models, behavioural experimentation was employed merely to illustrate that the model could detect objectively salient events, such as an animal call amidst pure noise. These studies had subjects choose the more salient of two presented scenes [33,35,38], and used a variety of natural environmental sounds as the salient events. Later models adapted more sophisticated paradigms where the background had a predictable structure, and the task was the detection of the salient event, which had a deviation from the predictable pattern [40,47], such as a violin note popping out of a stream of piano notes. While these efforts provide a structured way of investigating the precise characteristics of salience perception, their artificial structure limits their account of salience in realistic settings. Attempts at using unstructured natural soundscapes to probe the perception of auditory salience are being made, where subjects listen to real recordings and denote by an interface the time instances they think are salient or interesting [75]. However, unlike the visual domain, in which automatic eye saccades can be rapidly recorded for many scenes, the auditory method is not only much slower and inefficient, but suffers from conscious decision-making, and arguably does not represent purely bottom-up processing as well as its visual counterpart. An intuitive and objective ground-truth dataset for auditory salience would probably lead to a significant increase in modelling efforts, both in designing specialized computational systems that perform robust and efficient computations that can be incorporated into real-time naturalistic applications, and in comparing the performance of various mechanisms hypothesized to underlie neural attentional processing.

On the neural front, single-unit recordings from cats, monkeys and ferrets provide the most direct access to effects of attention on neural activity in the auditory system. While very informative about neural correlates of attentional modulation on brain networks, they are costly to perform, too invasive for human research and are limited in the amount of information that can be extracted about the intricate cortical networks engaged in auditory perception. They are also restricted to relatively simple or constrained behavioural paradigms that can be used to train animals in a laboratory setting.

The closest correlate to single-unit recordings for humans is electrocorticography (ECoG). Though highly invasive and applicable only to neurosurgery patients, this technique uses electrode grids placed on the exposed brain to investigate attentional modulation of cerebral cortex using rich and complex stimuli. By contrast, MEG and EEG offer non-invasive alternatives that are applicable to a more general population, even though they lack the spatial resolution of EcoG, and are more susceptible to artefacts. Unlike other behavioural measures, MEG and EEG also allow direct insights into neural processes without engaging explicit perceptual decision. However, analytic techniques need to be improved to balance the elimination of noise and preservation of neural information about attentional and perceptual states of subjects, especially in complex sound environments [76]. Further, particularly in studies of bottom-up attention, a common experimental design is such that the subject is instructed to ignore the auditory input and remain engaged in a visual task such as watching a silent film or reading a book. This paradigm is vulnerable to top-down attentional confounds in the absence of distracting auditory stimuli or sufficiently engaging visual tasks.

## 4. Applications of auditory attention models

Aside from providing important contributions to theoretical neuroscience, models of attention play a significant role in a large variety of engineering applications. Particularly, performance on tasks for which humans effortlessly outperform computers could be improved with attention mechanisms, where the attention component would act as a filter to guide computational resources to areas of maximum information, effectively reducing system noise by ignoring irrelevant parts of the scene. One such task is speech and sound recognition: although a trivial task to perform for humans, automatic recognition suffers from significant performance degradation in noisy environments. Some of the surveyed modelling studies have demonstrated various ways in which attentional mechanisms could work to improve existing recognition technologies. Feature-based approaches make use of the feature-extraction schemes of salience models as a way to get a perceptually informative representation of sound input. This representation can be used to detect prominent syllables from speech [34] or as an intermediate step for traditional speech feature extraction and recognition, or fed directly into a clustering mechanism for sound or emotion classification [77,78]. Top-down task–based adaptations have been incorporated in attention systems by modelling the attentional gain as weights in the classifier to optimize performance based on specific task goals [77,78], or as a separate cognitive model deciding which speaker to attend to among competing sources [79]. A more holistic attention mechanism has instead used the goal-directed adaptation framework of physiological STRFs as a pre-processing stage to speech recognition, by enabling the separation of the target speech stream from the distractor soundscape it is embedded in [80]. The attentional filter provides significant gain to the target speech while being robust to previously unseen noise types. This system was further generalized to use model STRFs optimized for the task, where STRFs are designed as two-dimensional filters, with their parameters estimated from training data [81]. Parametrizing the STRFs allowed for greater flexibility in implementing plasticity. While the authors demonstrated that this model resulted in better identification of speech in noise, the underlying framework can also be applied to a variety of auditory scene analysis problems by training STRFs for specific tasks.

The beneficial effect of attention has also been incorporated into numerous computational auditory models; we give some illustrative examples here. One computational system incorporated both bottom-up and top-down components to mimic human attentional orienting in a busy acoustic environment, allowing a soundscape designer to evaluate how the sound in planned urban environments might affect people [82]. A bottom-up attention mechanism specifically designed for efficient auditory surveillance demonstrated powerful detection of alarming sound events such as gunshots and screams in natural scenes [83]. It has been suggested [48] that attention models are of great importance for improvements in sonification systems aimed at converting information into sound (e.g. as aids for the blind). An integration of bottom-up and top-down modelling techniques replicating processes in the auditory pathway was demonstrated to improve sound localization in reverberant environments [84]. Auditory salience has been demonstrated to be an effective criterion for compression to reduce data size while retaining meaningful segments of large datasets of sound [85] and video [86]. Salience extraction has also been used as an abnormal sound detection mechanism for temporal signals, and generalized to lung sounds to use for finding medical abnormalities [87].

Finally, auditory attention models are an important component of audio–visual models and applications. In recent years, the necessity of incorporating auditory salience information in visual attention models is becoming increasingly recognized. This has led to the emergence of models using auditory salience direction to guide visual attention [89–90], along with audio–visual models where the two domains have equal weight in determining attentional orientation [91]. These models show better performance than visual-only salience models in predicting eye gazes in videos.

Mechanisms of multimodal attention are especially crucial in efficient designs of robotic systems [92] and brain–computer interfaces (BCIs). EEG being the most portable method by which brain signals can be recorded, models extracting cognitive information from EEG recordings are of particular significance for BCI systems. The surveyed stimulus reconstruction mechanisms that demonstrate the ability to detect who the subject is listening to have significant implications for powerful naturalistic BCIs. It is of particular interest that these methods are being optimized to use fewer electrodes and faster paradigms to achieve more portable, real-time interfaces [72]. Artificial intelligence systems need attentional filters to select sensory input to process in a goal-oriented manner, and to be able to adapt to unpredictable natural environments. Attention mechanisms have been modelled in various robot and machine-sensing applications [93,94]. However, these systems use platform-specific definitions of salience and attention, and do not have a direct correlate in the purely computational attention models described here. The computational modelling field has seen significant advances since these robotic sensory designs. Exploring the applicability of new models in robot perception can provide valuable direction to future models, and as computational architectures develop refined biologically plausible mechanisms, human-like robots will become a closer reality.
